# Haemolytic Uraemic Syndrome in an Adult Following Escherichia coli Sepsis: A Case Report

**DOI:** 10.7759/cureus.91417

**Published:** 2025-09-01

**Authors:** Ummulkhayr Yussuf, Christopher McManus

**Affiliations:** 1 General Internal Medicine, Southport and Ormskirk Hospital NHS Trust, Southport, GBR; 2 General Internal Medicine/Respiratory Medicine, Southport and Formby District General Hospital, Southport, GBR

**Keywords:** acute kidney injury, adult, e. coli, haemolytic uraemic syndrome (hus), sepsis, thrombocytopenia

## Abstract

This case report describes a previously healthy 43-year-old woman who developed haemolytic uraemic syndrome (HUS) following *Escherichia coli *sepsis. She presented with vomiting, diarrhoea, fever, right upper quadrant pain, and altered mental status after returning from travel to Bali and Australia. Initial investigations revealed stage 3 acute kidney injury, deranged liver function, anaemia, thrombocytopenia, and raised inflammatory markers. Blood and urine cultures confirmed *E. coli*, and she was treated with intravenous antibiotics and supportive care. A peripheral blood film showed features of microangiopathic haemolysis, consistent with HUS, although Shiga toxin testing was not reported in this case. Her condition gradually improved with conservative management. This case highlights the need to consider HUS in adults with *E. coli *sepsis, even without confirmed Shiga toxin, and the importance of prompt supportive treatment.

## Introduction

Haemolytic uraemic syndrome (HUS) is a form of thrombotic microangiopathy characterised by a triad of thrombocytopenia, microangiopathic haemolytic anaemia, and acute kidney injury [1]. It most commonly occurs in children following infection with Shiga toxin-producing *Escherichia coli *(STEC). In adults, HUS is less common and can present a diagnostic challenge, particularly when Shiga toxin is not identified. Adult cases may arise in the context of sepsis, malignancy, medications, or complement dysregulation. In particular, sepsis due to *E.coli *bacteraemia has been recognised as a potential trigger through endothelial injury and complement activation. Prompt recognition is essential to guide appropriate management and prevent long-term complications. This case describes an adult patient who developed HUS secondary to *E. coli *sepsis, highlighting the importance of considering this condition even in the absence of classical features.

## Case presentation

A 43-year-old woman presented with a three-day history of vomiting, diarrhoea, fever, the right upper quadrant abdominal pain. She also appeared confused, with a GCS of 14/15 on arrival. She had no known past medical history and had returned from travel to Australia and Bali 4 days prior to the presentation.

On admission, her National Early Warning Score (NEWS) was 8, with a respiratory rate of 38 breaths/min, heart rate of 132 beats/min, blood pressure of 95/58 mmHg, oxygen saturation of 97% on room air, and temperature of 37.8°C.

**Figure 1:**
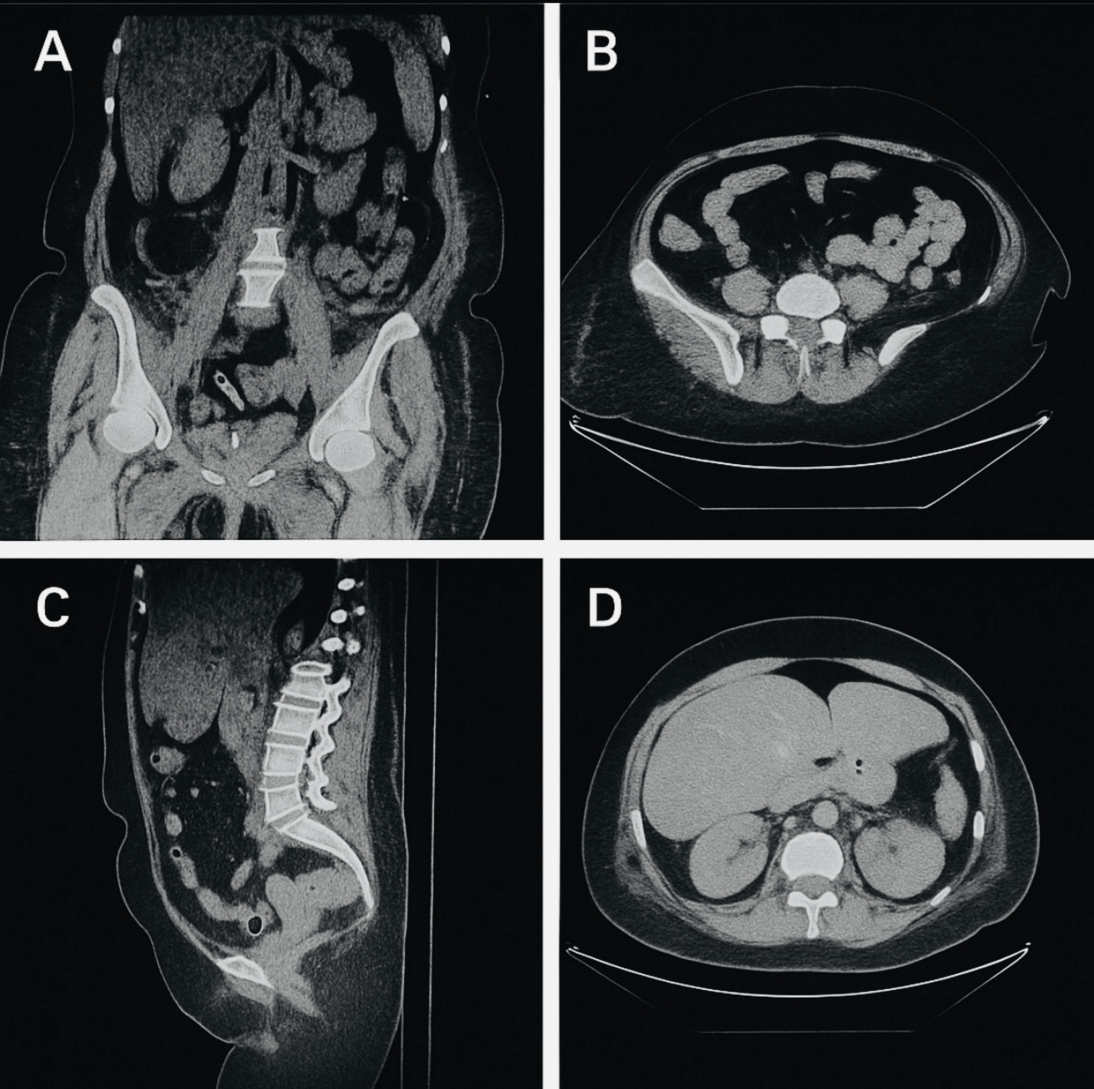
Contrast-enhanced CT images of the abdomen and pelvis A–D. Selected axial, coronal, and sagittal views. The scan showed diffuse hepatic steatosis, small bilateral pleural effusions with adjacent atelectatic/consolidative changes, and mild retroperitoneal fat stranding of uncertain significance. There was no hydronephrosis or abdominal collections. No significant lymphadenopathy or destructive bony lesions were identified.

Blood tests revealed stage 3 acute kidney injury, deranged liver function tests, anaemia with a haemoglobin of 103 g/L, thrombocytopenia (platelets 136 × 10⁹/L), and a significantly elevated C-reactive protein (CRP) of 342 mg/L, indicating significant inflammation and multiorgan dysfunction. She was started on empirical intravenous antibiotics, including amoxicillin, aztreonam, and metronidazole, for presumed sepsis of unknown source. A CT scan of the abdomen and pelvis showed no intra-abdominal source of infection, helping to guide further management (Figure 1). A chest infection was suspected due to the patient's worsening shortness of breath and consolidative changes on CT abdomen and pelvis.

**Figure 2:**
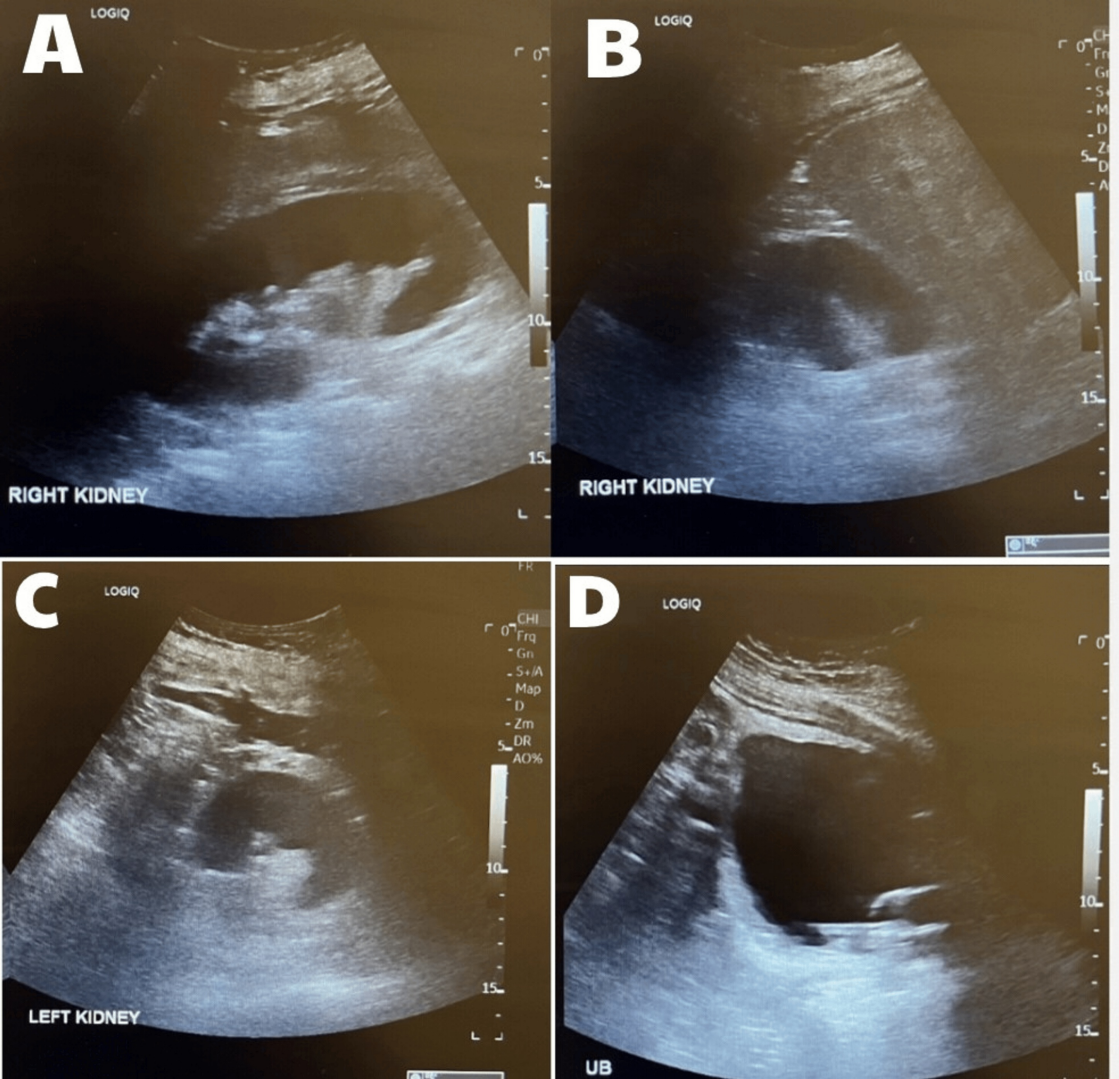
Ultrasound of the kidneys, ureters, and bladder (KUB) showing normal renal morphology with no evidence of hydronephrosis or obstructive uropathy Ultrasound of KUB demonstrating the right kidney (A, B), left kidney (C), and urinary bladder with catheter in situ (D).

By day four of admission, her haemoglobin had dropped to 74 g/L and platelets to 40 × 10⁹/L, without any signs of active bleeding. She received two units of packed red blood cells. Blood cultures and urine samples both grew *Escherichia coli*. Following microbiology advice, antibiotics were rationalised to intravenous aztreonam monotherapy, completed over 10 days. A renal ultrasound showed no structural abnormalities (Figure 2).

A broad infectious and autoimmune workup was performed. Stool tests were negative for *Clostridium difficile *and parasites. Viral screening for hepatitis (B, C), HIV, and malaria was all negative. Vasculitis screen, including antineutrophil cytoplasmic antibodies (ANCA), complement levels, and syphilis, was unremarkable.

A peripheral blood film revealed schistocytes, stomatocytes, target cells, and toxic granulation, in keeping with a microangiopathic haemolytic process. Thrombotic thrombocytopenic purpura (TTP) was considered; however, the absence of significant neurological features and the presence of marked renal impairment made HUS the more likely diagnosis.

Based on the clinical presentation and laboratory findings, a diagnosis of haemolytic uraemic syndrome (HUS), likely secondary to *E. coli *sepsis, was made following discussion with the haematology team.

Serial laboratory findings during admission are summarised in Table 1.

**Table 1:** Summary of Key Laboratory Findings During Hospital Admission WCC: White cell count; CRP: C-reactive protein; ALT: Alanine transaminase; GGT: Gamma-glutamyl transferase; LFTs: Liver function tests; g/L: grams per litre; mg/L: milligrams per litre; µmol/L: micromoles per litre; ×10⁹/L: billion cells per litre; U/L: units per litre

Investigation	First Day of Admission	Fourth Day of Admission	Last Day of Admission	Reference Range	Units
Haematology					
White cell count (WCC)	7.2	3	8.9	4.0–11.0	×10⁹/L
Neutrophils	6	2.3	6.7	2.0–7.5	×10⁹/L
Lymphocytes	0.8	0.5	1.7	1.0–3.5	×10⁹/L
Haemoglobin	103	74	85	115–165	g/L
Platelet count	136	40	730	150–450	×10⁹/L
C-reactive protein (CRP)	342	263	35	0-9	mg/L
Blood film findings	—	Schistocytes, stomatocytes, target cells, toxic granulation	—	—	—
Renal Function					
Serum creatinine	356	263	84	49–90 (female)	µmol/L
Liver Function Tests (LFTs)					
Alanine transaminase (ALT)	61	39	31	<35	U/L
Gamma-glutamyl transferase (GGT)	345	425	342	5–40	U/L
Total bilirubin	29	26	9	3–20	µmol/L
Albumin	31	24	31	35–50	g/L
Microbiology & Immunology					
Blood cultures	Escherichia coli	—	—	Negative	—
Urine cultures	Escherichia coli	—	—	Negative	—
Stool culture & Shiga toxin assay	Negative	—	—	Negative	—
Complement levels (C3, C4)	Normal	—	—	C3: 0.75–1.65, C4: 0.14–0.54	g/L
Vasculitis screen & viral serology	Negative	—	—	Negative	—

Her renal function and platelet count gradually returned to normal. However, at the time of discharge, her haemoglobin remained low (Table 1), though stable. She was discharged in a clinically stable condition and referred for outpatient follow-up.

## Discussion

While HUS is most commonly observed in children following infection with Shiga toxin-producing *Escherichia coli *(STEC), it can also occur in adults, where it is less frequently recognised and may present with atypical features [2,3].

In this case, a previously healthy adult developed HUS in the setting of confirmed *E. coli *bacteraemia and sepsis. Although Shiga toxin testing was not reported, the clinical course and laboratory findings supported a diagnosis of HUS. Similar cases have been described in published reports where adults developed HUS following *E. coli *infection, even when Shiga toxin was not confirmed [4,5].

The presence of schistocytes and other abnormal red blood cell morphologies on peripheral blood film supported the diagnosis of a microangiopathic haemolytic process. A significant drop in haemoglobin and platelets occurred in the absence of overt bleeding, and renal impairment was evident on admission. These features fulfilled the diagnostic criteria for HUS.

Management in this case was supportive, including intravenous antibiotics and blood transfusion. No targeted therapies, such as plasma exchange or complement inhibition, were administered, and the patient showed gradual improvement in renal function and platelet count. Supportive management remains the mainstay of treatment for typical or infection-associated HUS [6].

This case emphasises the importance of considering HUS in adults presenting with sepsis and unexplained thrombocytopenia, anaemia, and acute kidney injury. Although commonly associated with STEC infections in children, adult HUS can occur in a broader clinical context, including non-bloody diarrhoeal illness and culture-confirmed *E. coli *sepsis. Maintaining clinical awareness can facilitate early recognition and reduce the risk of long-term complications.

## Conclusions

This case highlights that HUS can occur in adults with *E. coli *sepsis even without confirmed Shiga toxin. Clinicians should remain alert to the possibility of HUS when evaluating adults with unexplained anaemia, thrombocytopenia, and acute kidney injury. In this case, early recognition and supportive care appeared to contribute to a favourable short-term outcome at discharge, but further studies are needed to better define long-term prognosis.
